# Outcomes after non-operative management of perforated diverticular disease: a population-based cohort study

**DOI:** 10.1093/bjsopen/zraa073

**Published:** 2021-04-22

**Authors:** A Adiamah, L Ban, H Otete, C J Crooks, J West, D J Humes

**Affiliations:** 1 National Institute for Health Research Nottingham Digestive Diseases Biomedical Research Unit, Nottingham University Hospitals NHS Trust, Nottingham, UK; 2 School of Medicine, Harrington building, University of Central Lancashire, Preston, UK; 3 Division of Epidemiology and Public Health, School of Medicine, University of Nottingham, City Hospital, Nottingham, UK

## Abstract

**Background:**

The management of perforated diverticular disease has changed in the past 10 years with a move towards less surgical intervention. This population-based cohort study aimed to define the risk of death and readmission following non-operative management of perforated diverticular disease.

**Methods:**

Patients diagnosed with perforated diverticular disease and managed without surgery were identified from the linked Clinical Practice Research Datalink and Hospital Episode Statistics data from 2000 to 2013. The outcomes were 1-year case fatality, readmissions, and surgery at readmission.

**Results:**

In total, 880 patients with perforated diverticular disease were managed without surgery, comprising 523 women (59.4 per cent). The 1-year case fatality rate was 33.2 per cent (293 of 880). The majority of deaths occurred in the first 90 days after the index admission, with a 90-day case fatality rate of 28.8 per cent. The 90-day survival rate varied by age, and was 97.2 per cent among those aged less than 65 years, compared with 85.0 per cent for those aged between 65 and 74 years, and 47.7 per cent in those at least 75 years old. Of 767 patients discharged from hospital, 250 (32.6 per cent) were readmitted (47 elective, 6.1 per cent; 203 emergency, 26.5 per cent) during a median of 1.6 (i.q.r. 0.1–3.9) years of follow-up, with similar proportions in each age category. In the first year of follow-up, only 5.1 per cent of patients required surgery, of whom 16 of 767 (2.1 per cent) required elective and 23 (3.0 per cent) emergency operation.

**Conclusion:**

Non-operative management of perforated diverticulitis in those aged less than 65 years is feasible and safe. Reintervention rates following conservative management were low across all age categories.

## Teaser

Among patients treated conservatively for perforated diverticulitis, 32.6 per cent of those discharged from hospital required emergency readmission during follow-up. Of these, only 3.0 per cent required emergency surgery. Reintervention rates after conservative management of perforated diverticular disease are reassuringly low.

## Resumen

**Antecedentes:**

El tratamiento de la enfermedad diverticular perforada ha cambiado en los últimos 10 años con una tendencia hacia un menor porcentaje de intervenciones quirúrgicas. Este estudio de cohortes de base poblacional tuvo como objetivo definir el riesgo de mortalidad y de reingreso después del tratamiento no quirúrgico de la enfermedad diverticular perforada (*diverticular disease*, DD).

**Métodos:**

Los pacientes diagnosticados con DD perforada y tratados sin cirugía se identificaron a partir de los datos vinculados del *Clinical Practice Research Datalink* y del *Hospital Episode Statistics* de 2000 a 2013. Las variables de interés eran la tasa de letalidad a 1 año, los reingresos y la cirugía durante el reingreso.

**Resultados:**

En total, 880 pacientes con DD perforada fueron tratados sin cirugía, entre ellos 523 mujeres (59,4%). La letalidad a 1 año fue del 33,2% (293/880). La mayoría de las muertes ocurrieron en los primeros 90 días después del primer ingreso con una letalidad a los 90 días del 28,8%. La supervivencia a los 90 días varió según la edad, con un 97,2% de supervivencia a los 90 días en los pacientes de menos de 65 años de edad en comparación con un 85,0% en los de 65 a 74 años y un 47,7 % en los mayores de 75 años. De 767 pacientes dados de alta del hospital, 250 (32,6%, 250/767) fueron reingresados, (47 programados (6,1%) y 203 reingresos urgentes (26,5%)) durante una mediana de 1,6 años de seguimiento (rango intercuartílico 0,1-3,9 años) con proporciones similares en cada categoría de edad. En el primer año de seguimiento solo el 5,1% de los pacientes requirieron cirugía, de los cuales 16/767 (2,1%) requirieron cirugía programada y 23/767 (3,0%) urgente.

**Conclusión:**

El tratamiento conservador de la diverticulitis perforada en pacientes de menos de 65 años de edad es factible y seguro. Las tasas de reintervención después de un tratamiento conservador fueron bajas en todas las franjas de edad.

## Introduction

Perforation is the most severe complication of diverticular disease and is becoming more common^1–3^. It is associated with significant mortality and surgical intervention is associated with significant morbidity^4^. An epidemiological study of Scottish National Health Service (NHS) hospitals reported an increase in annual emergency admissions for complicated diverticular disease from 22.9 per cent in 2000 to 27.1 per cent in 2010; this was associated with a decrease in the number of patients undergoing emergency surgery, suggesting that a greater proportion were being treated conservatively^5^. This change towards non-operative management is also acknowledged in recent UK professional guidelines^6^. Emerging evidence^7^ suggests that patients with diverticulitis and localized perforation can be managed successfully with a non-operative approach^7–11^. Evidence that informs this practice, however, reflects single-centre observational studies, small patient numbers, and often short-term outcomes^8–11^. Important patient-level risk factors and confounders for treatment failure, such as age, sex and co-morbidity, could not be explored with any precision, and there was also a high risk of selection bias.

There is a dearth of large population-based studies evaluating outcomes after conservative management of perforated diverticulitis. The natural history of patients with perforated diverticulitis who are managed conservatively in terms of disease recurrence, readmission rates, need for surgery, and mortality are poorly reported. This population-based cohort study evaluated these outcomes after non-operative management of perforated diverticular disease using healthcare data from England.

## Methods

This study was conducted and reported according to recommendations laid out in the STROBE checklist^12^. It received approval from the Independent Scientific Advisory Committee approval board, which provides scientific advice to the Medicines and Healthcare products Regulatory Agency (MHRA) on study design, and advised whether further approval was required from the Multi-centre Research Ethics Committee outside the MHRA’s current approval for observational studies (Protocol 16_226R).

### Study design

Two previously well characterized databases were used^13^^,^^14^. The Clinical Practice Research Datalink (CPRD) contains diagnostic and prescription data for over 14.1 million of the general population in the UK, with 3.4 million active patients contributing data^13^^,^^14^. Data are audited regularly with participating general practices, with quality checks to ensure that data are up to standard for research purposes. Hospital Episode Statistics (HES) is a data source containing detailed records of each episode of admitted patient care delivered in England either by NHS hospitals, or commissioned by the NHS but delivered in the independent sector^1^. Patient records in HES are coded using a combination of the ICD-10 codes for diagnoses at discharge, and OPCS-4 codes for the relevant procedure undertaken during that admission^1^^,^^13^^,^^14^.

### Validation

Because of the dependence on the accuracy of primary-care records, a two-stage approach described by Humes and colleagues^1^ was used to validate the case definition of perforated diverticulitis. This included a local audit of patients diagnosed with an ICD-10 code of K57.2 (diverticular disease of large intestine with perforation and abscess) and K57.8 (diverticular disease of intestine, part unspecified, with perforation and abscess). A sample of anonymized case records of patients classed as having perforated diverticulitis was also subsequently validated^1^. These definitions of perforated diverticulitis and diverticulitis associated with an abscess have been used in subsequent work in complicated diverticular disease^13^.

### Study group

All patients with an incident diagnosis of perforated diverticular disease were identified, who had a Read or Oxford Medical Information Systems code, or ICD-10 code for perforation due to colonic diverticular disease between 2000 and 2013. All patients with a code for colorectal cancer (C18–C21) were excluded. A case was defined as incident if no previous record of perforated colonic diverticular disease was entered in the patient’s record within 90 days of original registration at the patient’s general practice, as used elsewhere^15^. OPCS codes from HES data were used to identify patients with perforated diverticulitis who had undergone surgical resection. Patients without a surgical intervention during the index admission were considered to have been treated non-operatively and formed the study group.

### Co-variables

Age was subclassified as less than 65 years, 65–74 years, and 75 years or more. Co-morbidity was classified using the Charlson’s Co-morbidity Index^16^ as 0 or 1 or more, as identified from Read and ICD codes before the index admission. Smoking status was classified as never or ever smoked, based on CPRD records before operation. BMI before surgery was extracted from primary-care data, and categorized into four groups as: normal (BMI 18.5-24.9 kg/m^2^); underweight (BMI<18.5 kg/m^2^) and overweight (BMI 25-29.9 kg/m^2^); obese (BMI ≥30 kg/m^2^); or missing. Patients were followed up from the incident date of perforated diverticular disease diagnosis until date of death, end of available follow-up, or for 1 year. The 30-day, 90-day, and 1-year mortality rates were determined based on death registrations from Office for National Statistics data^1^. Duration of hospital stay and readmission data were determined for all patients who were alive at discharge (calculated as the difference between total number of patients and in-hospital deaths). Type of readmission at 1 year was classified as elective or emergency, and operative procedures at readmission were similarly grouped as emergency or elective colectomy.

### Statistical analysis

Demographic characteristics are reported using frequencies and percentages along with median age and duration of hospital stay. Case fatality was calculated as the number of deaths per each age-category at 30-days, 90-days and 1-year. A Cox proportional hazards model was fitted to estimate the hazard ratio for mortality. Patients were entered at the date of incident diagnosis of perforated diverticular disease, and were censored at the date on which death was recorded, they transferred out of the participating general practice, the practice was no longer deemed up to standard, or at 1 year after diagnosis, whichever was earliest. The analysis was adjusted for potential *a priori* confounders (age, sex, co-morbidity). The proportional hazards of the final models were checked using log-log plots. All analysis was performed using Stata/MP^®^ version 15 (StataCorp, College Station, Texas, USA).

## Results

**Fig. 1:**
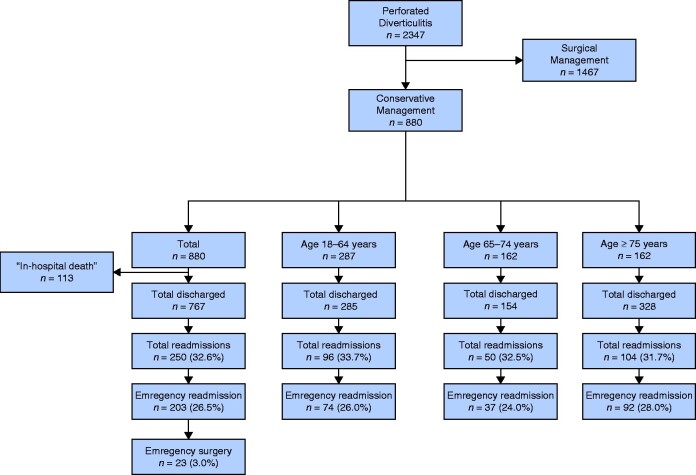
Study flow diagram

A total of 2347 patients were identified who had an incident diagnosis of perforated diverticular disease. Of these, 880 were managed without surgery and formed the study population (*Fig. 1*). There were 523 women (59.4 per cent). Median age was 75 (i.q.r. 59–85) years. The proportion of the population aged 65 years or older was 67.4 per cent (593 patients). Demographic characteristics of patients in the study are summarized in *Table 1* and *Table S1*.

**Table 1:** Baseline demographics of patients with conservatively managed perforated diverticular disease

	Entire cohort	Survivors	Non-survivors	***P*** ^†^
	(*n* = 880)	(*n* = 767)	(*n* = 113)	
**Age (years)**				< 0.001
18–64	287 (32.6)	285 (37.2)	–*	
65–74	162 (18.4)	154 (20.1)	–*	
≥ 75	431 (49.0)	328 (42.8)	103 (91.2)	
**Sex ratio (M : F)**	357 : 523	327 : 440	30 : 83	0.001
**Co-morbidity**				0.008
None	412 (46.8)	373 (48.6)	39 (34.5)	
≥1	468 (53.2)	394 (51.4)	74 (65.5)	
**BMI category**				0.017
Normal weight	248 (28.2)	218 (28.4)	30 (26.5)	
Overweight or underweight	277 (31.5)	238 (31.0)	39 (34.5)	
Obese	236 (26.8)	216 (28.2)	20 (17.7)	
Missing	119 (13.5)	95 (12.4)	24 (21.2)	
**Smoking status**				
Non-smoker	318 (36.1)	267 (34.8)	51 (45.1)	0.007
Ever smoker	529 (60.1)	475 (61.9)	54 (47.8)	
Missing	33 (3.8)	25 (3.3)	8 (7.1)	

Values in parentheses are percentages. *Cell count too low to report. †Chi square test.

### In-hospital mortality

The in-hospital mortality rate was 12.8 per cent (113 of 880); patients aged 75 years or older accounted for 103 in-hospital deaths (91.2 per cent). The total number of patients who survived to discharge and were at risk of readmission was 767.

### Duration of hospital stay

The overall median total duration of stay was 4 (i.q.r. 1–8) days. There was no significant difference in median length of stay by age categories.

### Case fatality and survival at 30 days, 90 days, and 1 year

The 30-day survival rate was 98.3 (95 per cent c.i. 95.8 to 99.3) per cent overall, and 57.7 (52.9 to 62.2) per cent among those aged 75 years or more. The case fatality rate at 30 days was 1.7 per cent in those younger than 65 years, representing an 80 per cent reduction in risk of death compared with those aged 65–74 years (adjusted HR 0.17, 95 per cent c.i. 0.06 to 0.46). Among patients aged 75 years or more, there was a nearly five-fold increase in risk of death at 30 days compared with those aged 65–74 years (case fatality rate 42.2 per cent; adjusted HR 4.64, 95 percent c.i. 2.81 to 7.64) (*Table 2*)

**Table 2:** Survival and adjusted hazard ratios for death at 30 days, 90 days, and 1 year after management of perforated diverticulitis

	Case fatality rate*	Survival (%)^†^	Unadjusted HR^†^	Adjusted HR^†‡^
**30 days**				
18–64	5 of 287 (1.7)	98.3 (95.8, 99.3)	0.16 (0.06-0.43)	0.17 (0.06-0.46)
65–74	17 of 162 (10.5)	89.5 (83.6, 93.3)	1.00 (reference)	1.00 (reference)
≥ 75	182 of 431 (42.2)	57.7 (52.9, 62.2)	4.86 (2.96-8.00)	4.64 (2.81-7.64)
**90 days**				
18–64	8 of 287 (2.8)	97.2 (94.4, 98.6)	0.18 (0.08-0.39)	0.19 (0.08-0.42)
65–74	24 of 162 (14.8)	85.0 (78.5, 89.7)	1.00 (reference)	1.00 (reference)
≥ 75	222 of 431 (51.5)	47.7 (42.9, 52.4)	4.50 (2.95-6.86)	4.31 (2.82-6.58)
**1 year**				
18–64	10 of 287 (3.5)	96.3 (93.3, 98.0)	0.17 (0.09-0.36)	0.19 (0.09-0.39)
65–74	30 of 162 (18.5)	81.0 (73.9, 86.3)	1.00 (reference)	1.00 (reference)
≥ 75	253 of 431 (58.7)	39.2 (34.5, 43.8)	4.39 (3.00-6.41)	4.17 (2.85-6.10)

Values in parentheses are *percentages and †95 per cent confidence intervals. Hazard ratios (HRs) were calculated using Cox proportional hazards model. ‡Adjusted for age, sex, and co-morbidity (defined using Charlson’s Co-morbidity Index).

**Fig. 2:**
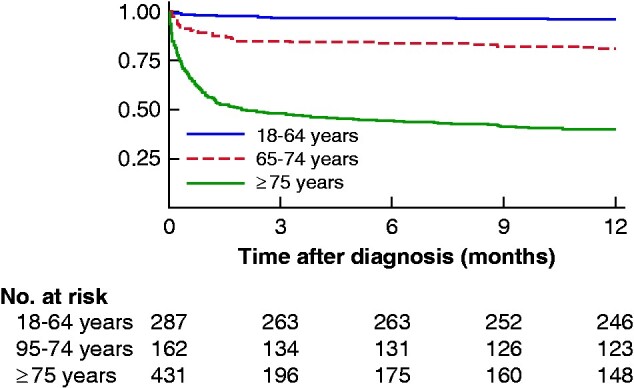
Kaplan–Meier survival estimates at 1 year by age category

A similar relationship was seen at 90 days. The Kaplan–Meier survival curve confirmed that the majority of deaths occurred within 90 days of the initial admission (*Fig. 2*). At 1 year, the survival rate was 96.3 (93.3 to 98.0) per cent in those younger than 65 years, but only 39.2 (34.5 to 43.8) among those aged 75 years and over (*Table 2* and *Table S2*).

No relationship was found between BMI and mortality at any time point.

### Readmission

**Fig. 3:**
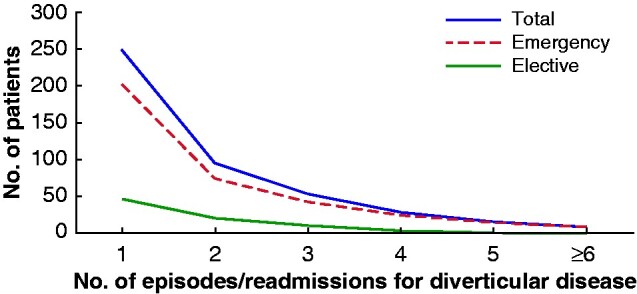
Total diverticular disease-related readmissions during total follow-up. Median follow-up was 1.6 years and longest follow-up 14.2 years. A total of 250 patients were readmitted during total follow-up. 154 patients were readmitted only once, and nine patients had six or more readmissions during total follow-up.

A total of 767 of 880 patients survived to discharge. One-third of these patients (250 of 767, 32.6 per cent) were readmitted during a median of 1.6 (i.q.r. 0.1–3.9) years of follow-up, including 47 elective readmissions (18.8 per cent) and 203 (81.2 per cent) emergency readmissions for recurrence or complications related to diverticular disease. Of all patients readmitted, 154 of 250 (61.6 per cent) were admitted once (*Fig. 3*).

A similar proportion of patients was readmitted across the different age categories during the entire follow-up period. Some 96 of 285 patients (33.7 per cent) were readmitted in the 18–64-year group, 50 of 154 (32.5 per cent) in the 65–74-year group, and 104 of 328 (31.7 per cent) aged over 75 years.

In multivariable cox regression analysis, only co-morbidity was associated with an increased risk of readmission at 1 year of follow-up (*Table S3*). Importantly, no relationship was identified between readmission and BMI.

### First-year readmission, operative procedures, and duration of stay

In the first year after the index admission, 173 of the 767 patients who survived to discharge were readmitted (22.6 per cent). This included 65 of 285 patients (22.8 per cent) aged 18–64 years, 28 of 154 (18.2 per cent) in the 65–74-year group, and 80 of 328 (24.4 per cent) aged 75 years or more (*Table 3*).

Emergency admission was the predominant type of readmission. There were 50 emergency and 15 elective readmissions in the 18–64-year group, 21 emergency and seven elective admissions in the 65–74-year group, and 70 emergency and 10 elective admissions among patients aged 75 years or more.

In the first year of follow-up, 16 of 767 patients (2.1 per cent) underwent emergencies elective surgery and 23 (3.0 per cent) emergency surgery during readmission. Among those aged 18–64 years, 10 of 285 (3.5 per cent) underwent elective surgery and another 11 (3.9 per cent) had an emergency operation. Owing to the limited number of operative interventions in the two older groups, the data are reported together for 482 patients aged at least 65 years. Of these, only six (1.2 per cent) underwent elective surgery and 12 (2.5 per cent) emergency surgery in the first year of follow-up (*Table 3*). Among these 18 patients, 11 operations were Hartmann’s procedures and none were reversed.

**Table 3:** Readmissions and surgery during the first year after index admission with conservatively managed perforated diverticulitis

	18–64 years	65–74 years	≥ 75 years	Total
**First-year readmissions**	*n* = 285	*n* = 154	*n* = 328	*n* = 767
Total	65 (22.8)	28 (18.2)	80 (24.4)	173 (22.6)
Elective readmission	15 (5.3)	7 (4.5)	10 (3.0)	32 (4.2)
Emergency readmission	50 (17.5)	21 (13.6)	70 (21.3)	141 (18.4)
**First-year surgery at readmission**	*n* = 285	*n* = 482^*^		*n* = 767
Total	21 (7.4)	18 (3.7)		39 (5.1)
Elective surgery	10 (3.5)	6 (1.2)		16 (2.1)
Emergency surgery	11 (3.9)	12 (2.5)		23 (3.0)

*Owing to reporting guidelines regarding cell numbers, the total number of operations in the two older groups have been combined to allow reporting of operative interventions in these patients.

Median duration of stay on readmission (with or without surgery) was 6 (i.q.r. 2–10) days overall, 5 (2–9) days for patients admitted electively and 6 (3–10) days for those admitted as an emergency.

## Discussion

In patients younger than 65 years, conservatively management of perforated diverticulitis is safe and associated with a low risk of death both in the short (30 days) and longer (1 year) term. The in-hospital mortality rate of 12.8 per cent was predominantly accounted for by those aged 75 years (91.2 per cent of in-hospital deaths) and these patients had a proportionally higher case fatality rate at 30 days, 90 days, and 1 year. These results suggest that those of advanced age with co-morbidity may have been considered unfit for surgery and managed non-operatively on that basis. The data sources used did not include information on this aspect of decision-making.

One-third of all patients who survived the index admission were readmitted during the entire follow-up and this did not vary by age. The majority of readmissions occurred within the first year of index admission and 81.5 per cent of these readmissions (141 of 173) were as an emergency, although only small proportion of patients (5.1 per cent) needed surgery as a result of readmission in the first year. This suggests that, in the short term, conservative management of locally perforated diverticular disease is safe, but the patient must be informed of the risk of subsequent readmissions, especially in the first year.

Two population-based studies^5^^,^^17^ have reported a trend away from surgical intervention in the management of perforated diverticular disease. The proportion of patients undergoing surgical intervention for emergency admissions with diverticular disease fell from a peak of 16.3 per cent in 2002 to 11.5 per cent in 2010 in Scotland^5^, and from 17 to 14 per cent in the USA^17^. This decline in use of surgery and increase in conservative management was not thought to be a result of less severe disease, but considered to reflect better supportive care and more accurate selection of patients, potentially influenced by the availability of and improvements in high-resolution CT^5^. The majority of nationwide clinical guidelines now recommend CT to confirm and grade the severity of acute diverticular disease^18^ and to guide management^19^.

Four observational studies have suggested non-operative management of perforated diverticulitis to be safe. These small single-centre studies had total numbers of 39^9^, 64^10^, 132^8^, and 136 patients^11^ respectively. Follow-up was limited, and none explored the impact of age and co-morbidity on findings. One study^10^, however, found that ASA grade III and IV, and distant location of air, were the two predictors of failure of non-operative treatment, whereas another^11^ reported a high success rate of conservative treatment in patients with perforated diverticulitis and pneumoperitoneum who were haemodynamically stable. Sallinen and colleagues^8^ described the feasibility of conservative management in haemodynamically stable patients with pericolic extraluminal air or a small amount of distant intraperitoneal air in the absence of clinical diffuse peritonitis. No study reported on readmission or subsequent need for surgical intervention.

A study from New Zealand reported on patterns of readmission after an initial admission with complicated or uncomplicated diverticulitis, and found an overall recurrence rate of 24 per cent in patients with complicated diverticulitis (abscess and perforation), with the majority of readmissions occurring in the first year after index presentation^20^. As in the present study, no difference was found in recurrence between young (aged less than 50 years) and older patients^20^.

The present study was reliant on coding databases with a risk of misclassification. This approach also did not allow a useful predictor, such as extent of intraperitoneal contamination, to be considered as a factor determining the success of non-operative management^7^^,^^19^. BMI has been shown recently to be associated with an increased risk of perforated diverticulitis and readmission; however, in the present study, although a majority (60 per cent) of patients had an abnormal BMI (overweight or obese), this was not predictive of death or readmission in Cox regression analysis. This could have been influenced by the incompleteness of BMI data (13.5 per cent missing data).

The methodology used here, however, is based on a previously validated approach^1^ for confirmation of the diagnosis of perforated diverticulitis in the HES and CPRD linked data sets. The ability to describe mortality rates by age group, risk of readmission, and low rates of surgery should readmission be necessary, provides reassurance to surgeons and better information to patients in shared decision-making.

## Funding

The work was funded by CORE grant awarded to D.J.H.

*Disclosure:* The authors declare no conflict of interest. The funders had no role in the study design, data analysis or presentation of findings.

## Supplementary material

Supplementary material is available at *BJS Open* online.

## Supplementary Material

zraa073_Supplementary_Data
