# Supplementary material for: Outcomes after non-operative management of perforated diverticular disease: a population-based cohort study
Source: BJS Open. 2021 Apr 22;5(2):zraa073. doi: 10.1093/bjsopen/zraa073 (PMC8062256; doi:10.1093/bjsopen/zraa073)
Supplement: zraa073_Supplementary_Data [file zraa073_supplementary_data.docx]

Supplementary Table A: Baseline demographics by age categories

|  | **18 – 64 years** | | **65-74 years** | | **≥75** | |  |
| --- | --- | --- | --- | --- | --- | --- | --- |
|  | Frequency  (n=287) | Percentage  (32.61%) | Frequency  (n=162) | Percentage  (18.41%) | Frequency  (n=431) | Percentage  (48.98%) | P-value |
| **Gender** |  |  |  |  |  |  |  |
| Male | 164 | 57.14 | 69 | 42.59 | 124 | 28.77 | <0.0001 |
| Female | 123 | 42.86 | 93 | 57.41 | 307 | 71.23 |  |
| **Comorbidity** |  |  |  |  |  |  |  |
| No comorbidity | 186 | 64.81 | 69 | 42.59 | 157 | 36.43 | <0.0001 |
| ≥1 Comorbidity | 101 | 35.19 | 93 | 57.41 | 274 | 63.57 |  |
| **Body Mass Index (Kg/m^2^)** |  |  |  |  |  |  |  |
| Normal Weight | 76 | 26.48 | 54 | 33.33 | 118 | 27.38 | <0.0001 |
| Overweight | 83 | 28.92 | 51 | 31.48 | 143 | 33.18 |  |
| Obese | 107 | 37.28 | 47 | 29.01 | 82 | 19.03 |  |
| Missing | 21 | 7.32 | 10 | 6.17 | 88 | 20.42 |  |
| **Smoking Status** |  |  |  |  |  |  |  |
| Non-Smoker | 90 | 31.36 | 45 | 27.78 | 183 | 42.46 | <0.0001 |
| Ever Smoker | 194 | 67.60 | 111 | 68.52 | 224 | 51.97 |  |
| Missing | * | * | 6 | 3.70 | 24 | 5.57 |  |

Legend:

*denotes cell count – is too low to report.

Supplementary Table B: Univariate and Multivariate Cox regression analysis of mortality at 1-year

|  |  | |  | |  |  |  |
| --- | --- | --- | --- | --- | --- | --- | --- |
|  | | **Univariate** | |  | | **Multivariate** |  |
| **Gender** | | HR | | 95% CI | | HR | 95% CI |
| Male | | ref | |  | |  |  |
| Female | | 1.95 | | 1.51-2.51 | | 1.28 | 0.99-1.65 |
| **Age** | |  | |  | |  |  |
| 18-64 | | 0.17 | | 0.09-0.36 | | 0.19 | 0.09-0.39 |
| 65-74 | | ref | |  | | ref |  |
| ≥75 | | 4.39 | | 3.00-6.41 | | 4.17 | 2.85-6.11 |
| **BMI cat** | |  | |  | |  |  |
| normal | | ref | |  | |  |  |
| Under/overweight | | 1.02 | | 0.75-1.39 | |  |  |
| obese | | 0.73 | | 0.52-1.03 | |  |  |
| missing | | 2.39 | | 1.73-3.31 | |  |  |
| **Smoking Status** | |  | |  | |  |  |
| Non-smoker | | ref | |  | |  |  |
| Ever smoked | | 0.72 | | 0.57-0.92 | |  |  |
| Missing | | 2.19 | | 1.39-3.45 | |  |  |
| **Comorbidity** | |  | |  | |  |  |
| 0 | | ref | |  | |  |  |
| 2 | | 2.02 | | 1.59-2.58 | | 1.32 | 1.03-1.69 |

- ^*Multivariate analysis adjusted for the factors significant in univariate analysis, age, gender, comorbidity as defined by Charlson’s comorbidity in^

Supplementary Table C: Univariate and Multivariate Cox regression analysis of readmission at 1-year

|  |  | |  | |  |  |  |
| --- | --- | --- | --- | --- | --- | --- | --- |
|  | | **Univariate** | |  | | **Multivariate** |  |
| **Gender** | | HR | | 95% CI | | HR | 95% CI |
| Male | | ref | |  | | ref |  |
| Female | | 1..09 | | 0.85-1.40 | | 1.10 | 0.86-1.71 |
| **Age** | |  | |  | |  |  |
| 18-64 | | 0.98 | | 0.69-1.38 | | 1.06 | 0.74-1.50 |
| 65-74 | | ref | |  | | ref |  |
| ≥75 | | 1.21 | | 0.86-1.69 | | 1.21 | 0.86-1.71 |
| **BMI cat** | |  | |  | |  |  |
| normal | | ref | |  | | ref |  |
| Under/overweight | | 1.10 | | 0.80-1.50 | | 1.07 | 0.78-1.47 |
| obese | | 1.03 | | 0.74-1.43 | | 1.03 | 0.74-1.44 |
| missing | | 0.88 | | 0.56-1.47 | | 0.90 | 0.53-3.24 |
| **Smoking Status** | |  | |  | |  |  |
| Non-smoker | | ref | |  | | ref |  |
| Ever smoked | | 1.25 | | 0.95-1.64 | | 1.26 | 0.95-1.68 |
| Missing | | 1.15 | | 0.50-2.65 | | 1.31 | 0.53-3.24 |
| **Comorbidity** | |  | |  | |  |  |
| 0 | | ref | |  | | ref |  |
| 2 | | 1.39 | | 1.08-1.78 | | 1.32 | 1.01-1.71 |

*_As only, comorbidity significant in univariate analysis, mutually adjusted for all variables_*.
